# Left ventricular transmural pressure and ventilatory inefficiency during exercise in advanced heart failure

**DOI:** 10.1093/eschf/xvag247

**Published:** 2026-10-07

**Authors:** Barkin Kultursay, Seda Tanyeri Uzel, Murat Karacam, Seref Berk Tuncer, Azmican Kaya, Halit Eminoglu, Suleyman Cagan Efe, Gulumser Sevgin Halil, Cem Dogan, Deniz Mutlu, Mehmet Kaan Kirali, Rezzan Deniz Acar, Emre Aslanger

**Affiliations:** Department of Cardiology, Tunceli State Hospital, Cumhuriyet Mah. 552. Sokak No: 2, 62000 Tunceli/Merkez, Türkiye; Department of Cardiology, Kartal Kosuyolu High Specialization Education and Research Hospital, Istanbul, Turkiye; Department of Cardiology, Bitlis State Hospital, Bitlis, Turkiye; Department of Cardiology, Kartal Kosuyolu High Specialization Education and Research Hospital, Istanbul, Turkiye; Department of Cardiology, Kartal Kosuyolu High Specialization Education and Research Hospital, Istanbul, Turkiye; Department of Cardiology, Kartal Kosuyolu High Specialization Education and Research Hospital, Istanbul, Turkiye; Department of Cardiology, Kartal Kosuyolu High Specialization Education and Research Hospital, Istanbul, Turkiye; Department of Cardiology, Kartal Kosuyolu High Specialization Education and Research Hospital, Istanbul, Turkiye; Department of Cardiology, Kartal Kosuyolu High Specialization Education and Research Hospital, Istanbul, Turkiye; Department of Interventional Cardiology, Mount Sinai Hospital, New York City, NY, USA; Department of Cardiovascular Surgery, Kartal Kosuyolu High Specialization Education and Research Hospital, Istanbul, Turkiye; Department of Cardiology, Kartal Kosuyolu High Specialization Education and Research Hospital, Istanbul, Turkiye; Department of Cardiology, Basaksehir Pine and Sakura City Hospital, Istanbul, Turkiye

**Keywords:** Cardiopulmonary exercise testing, Heart failure, Haemodynamics, Ventilatory efficiency

## Abstract

**Background:**

Baseline intracavitary haemodynamic measurements frequently demonstrate an inconsistent relationship with exertional performance. This may reflect an incomplete interpretation of left ventricular (LV) filling pressures. LV transmural pressure difference (ΔP_TM_ = LV end-diastolic pressure [LVEDP]—right atrial pressure [RAP]) integrates LV filling pressure with right ventricular/pericardial constraint and may provide a composite representation of resting haemodynamic conditions. Ventilatory efficiency, assessed by the minute ventilation-carbon dioxide production (V_E_/VCO_2_) slope during cardiopulmonary exercise testing (CPET), is a leading representation of cardiopulmonary reserves and a powerful prognostic marker in heart failure (HF). Whether ΔP_TM_ is associated with exercise ventilatory efficiency beyond conventional resting filling pressure measurements remains unclear.

**Methods:**

We retrospectively studied 422 patients with advanced HF (LVEF ≤25%) who underwent right-heart catheterization and CPET. ΔP_TM_ was analysed both continuously and by tertiles. The primary outcome was V_E_/VCO_2_ slope; peak VO_2_ was a secondary outcome. Bayesian multivariable regression models were adjusted using a directed acyclic graph-derived confounder set. Sensitivity analyses compared ΔP_TM_ with LVEDP and RAP modelled individually and jointly.

**Results:**

Among 422 patients (mean age 51.3 ± 10.8 years; 62 [14.7%] female), higher ΔP_TM_ was independently associated with a lower V_E_/VCO_2_ slope (posterior mean β = −0.34 per mmHg; 95% credible interval [CrI] −0.58 to −0.11; 99.7% posterior probability of a negative association). Unadjusted V_E_/VCO_2_ slope decreased from 40.2 (31.7–67.1) in the lowest ΔP_TM_ tertile to 34.9 (30.6–44.8) in the highest tertile (*P* = .029). The adjusted posterior predictive difference between the highest and lowest ΔP_TM_ tertiles was −4.42 units (95% CrI −7.85 to −0.62). Following multivariable adjustment, ΔP_TM_ showed no significant association with peak VO_2_ (posterior mean β = +0.06; 95% CrI −0.02 to +0.14). Neither LVEDP nor RAP, modelled individually or jointly, demonstrated independent associations with ventilatory efficiency.

**Conclusion:**

Resting ΔP_TM_ was inversely associated with the V_E_/VCO_2_ slope during exercise, whereas no independent association was observed with peak VO_2_. Compared with individual left- or right-sided filling pressures, ΔP_TM_ provides a parsimonious summary of the balance between left-sided distending pressure and right-sided constraint associated with ventilatory efficiency in advanced HF.

## Graphical Abstract

**Graphical Abstract:**
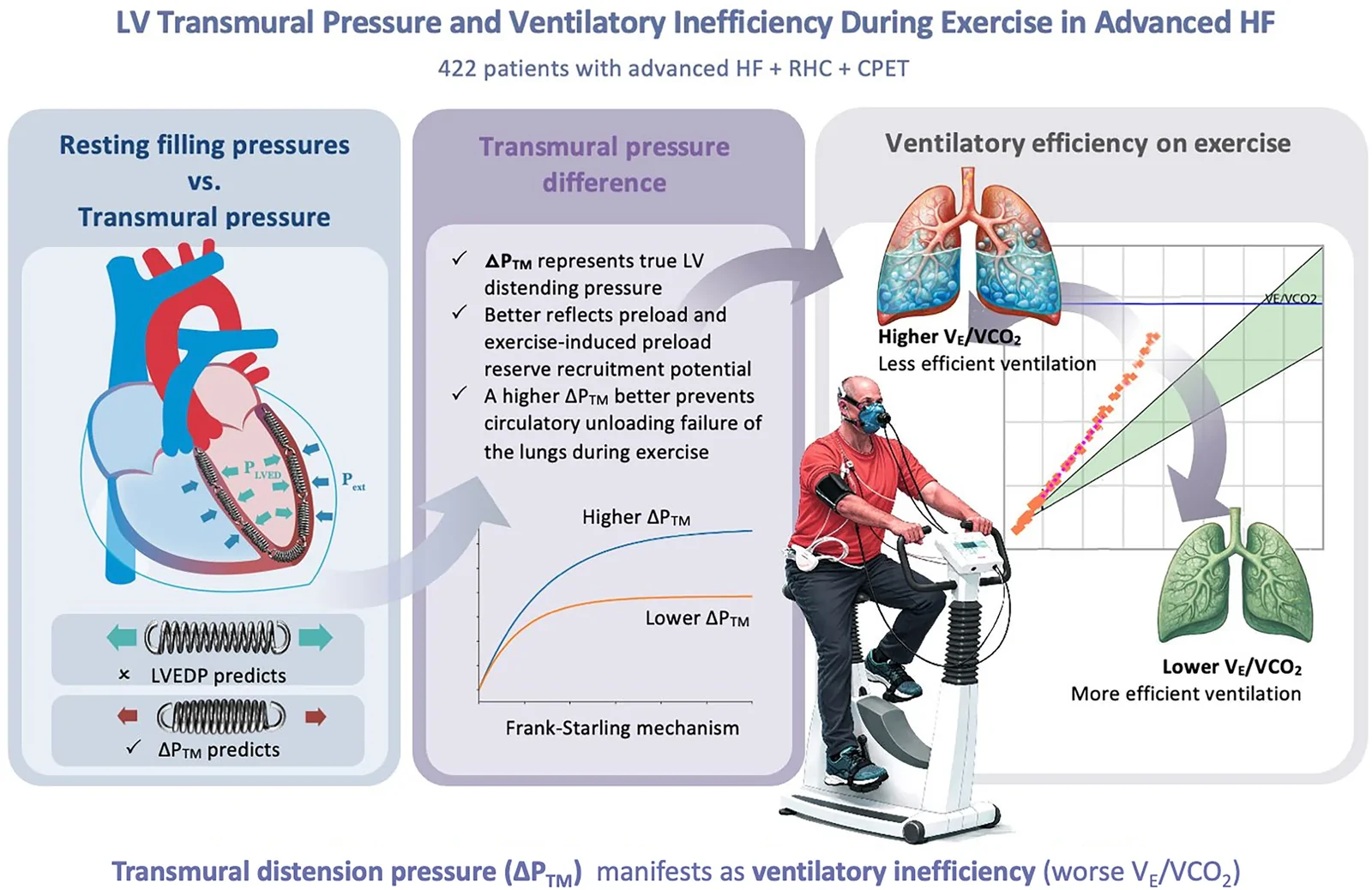

## Introduction

The interaction between baseline haemodynamics and exercise-related cardiopulmonary responses in heart failure (HF) is complex and incompletely understood. Although invasive filling pressures, such as left ventricular end-diastolic pressure (LVEDP), pulmonary capillary wedge pressure (PCWP), and right atrial pressure (RAP), are central to the assessment of congestion and loading conditions, their relationship with functional limitation is often inconsistent. This discordance is particularly relevant in advanced systolic HF, where ventricular interdependence, right-sided loading, pulmonary vascular dysfunction, and pericardial constraint may alter the physiological meaning of conventional intracavitary pressures.

This discrepancy may, in part, reflect an incomplete physiologic interpretation of conventional resting measurements. Experimental studies have shown that left ventricular (LV) wall stretch is determined not by intracavitary pressure alone, but by transmural pressure difference (ΔP_TM_) after accounting for extrinsic pericardial constraint.^1,2^ Because pericardial pressure closely approximates RAP, LV transmural pressure can be clinically estimated as ΔP_TM_ = LVEDP-—RAP.^3–7^ This physiologically grounded metric has therefore been proposed as a clinically useful estimate of LV distending pressure relative to intracavitary pressure alone, particularly in patients with elevated right-sided pressures.^8–10^

Cardiopulmonary exercise testing (CPET) provides complementary information by assessing the integrated cardiovascular, pulmonary, and peripheral response to exertion. Among CPET-derived parameters, the minute ventilation-carbon dioxide production slope (V_E_/VCO_2_ slope) is widely accepted as a sensitive marker of ventilatory inefficiency and one of the most powerful prognostic indicators in HF.^11–14^ The V_E_/VCO_2_ slope reflects several downstream abnormalities, including ventilation–perfusion mismatch, increased dead-space ventilation, heightened ventilatory drive, and impaired circulatory response during exertion.^11,12^ These processes may be influenced not only by LV filling pressure but also by right-sided loading and cardiopulmonary coupling, making V_E_/VCO_2_ slope a potentially relevant functional correlate of ΔP_TM_.

Prior studies have not demonstrated a consistent association between baseline haemodynamic parameters and the VE/VCO_2_ slope; however, the role of ΔP_TM_ has not been explored. Because ΔP_TM_ integrates left-sided filling pressure with right-sided/pericardial constraint, it may provide a composite representation of resting haemodynamic conditions associated with ventilatory efficiency.

We therefore examined the association between ΔP_TM_ and V_E_/VCO_2_ slope in patients with advanced systolic HF who underwent invasive haemodynamic assessment and CPET. We further sought to compare the association of ΔP_TM_ with ventilatory efficiency against those of its individual pressure components (LVEDP and RAP).

## Methods

### Study population

Consecutive patients admitted to the heart transplantation outpatient clinic between 2021 and 2024, who underwent comprehensive echocardiographic examination, CPET, and right heart catheterization (RHC), were retrospectively included in this study. To define a clinically homogeneous cohort with advanced systolic HF, patients with a left ventricular ejection fraction (LVEF) >25%, severe chronic pulmonary disease, or incomplete or missing CPET or RHC data were excluded. Transthoracic echocardiography was performed within one week prior to RHC and CPET. Demographic characteristics and laboratory data were obtained from electronic hospital records. The study was approved by the local ethics committee (approval no: 2026/03/1371), which waived the requirement for written informed consent due to the retrospective, observational design of the study, and the research was conducted in accordance with the Declaration of Helsinki.

### Echocardiography

Comprehensive echocardiographic examinations were performed using the EPIQ CVx ultrasound system (version 9.0.5; Philips Medical Systems, USA) equipped with S5-1 and X5-1 transducers. All studies were conducted in accordance with current echocardiographic guidelines by a single experienced cardiologist.^15,16^ LVEF was calculated using the biplane method of disks summation (modified Simpson’s rule). Right ventricular systolic function was assessed by tricuspid annular plane systolic excursion (TAPSE), fractional area change, and the peak systolic velocity of the lateral tricuspid annulus (S′, measured by tissue Doppler imaging). Mitral and tricuspid regurgitation were assessed using the integrative, multiparametric approach recommended by the American Society of Echocardiography and European Association of Cardiovascular Imaging guidelines (including qualitative, semiquantitative, and quantitative parameters when available). A four-category scale (none, mild, moderate, severe) was applied for consistency. Pulmonary artery systolic pressure was estimated by summing the pressure gradient derived from the peak velocity of tricuspid regurgitation (via the simplified Bernoulli equation) and the estimated RAP, based on inferior vena cava diameter and its respiratory variation.

### Exercise testing

A maximal exercise test was as assessed by a respiratory exchange ratio ≥1.05 and a modified Borg Scale ≥8. The maximal CPET was performed using a continuous, incremental treadmill protocol based on an individualized ramp design, conducted on a JAEGER Vyntus CPX system (Vyaire Medical, Germany). Oxygen uptake was measured breath by breath using an automated system, with data collected at rest, during graded exercise, and throughout a 2-min recovery period. The rate of oxygen consumption (VO_2_), carbon dioxide output (VCO_2_), and respiratory exchange ratio were computed and averaged every 10 s. Peak VO_2_ was defined as the highest 10-s averaged VO_2_ during the last stage of the exercise test. The V_E_/VCO_2_ slope was calculated via least-squares linear regression using breath-by-breath data obtained from the onset of loaded exercise to the respiratory compensation point (RCP). The RCP was identified by a progressive increase in the V_E_/VCO_2_ equivalent accompanied by a distinct decrease in end-tidal CO_2_ (P_ET_CO_2_) downstream of the ventilatory threshold. Resting, unloaded warm-up, post-RCP, and recovery data points were systematically excluded from the linear regression analysis. Blood pressure was measured periodically before and throughout the test every 3 min and in the recovery phase.

### Cardiac catheterization

RHC was performed through the right internal jugular or femoral vein using a 7-Fr Swan-Ganz catheter (Edwards Lifesciences, Irvine, California, USA). In patients who underwent concomitant diagnostic left heart catheterization, femoral arterial access was additionally obtained, and LVEDP was measured directly using a pigtail catheter advanced retrogradely across the aortic valve into the left ventricle. CPET and RHC were performed on separate occasions within a maximum window of 1 week (median interval: 3 days). Because these procedures were non-concurrent, resting cardiac output during catheterization was estimated using the indirect Fick method with assumed oxygen consumption, as direct breath-by-breath gas analysis was available only during the subsequent CPET. Direct invasive LVEDP measurements were available in 390 patients (92.4%). In the remaining 32 patients (7.6%), in whom left heart catheterization was not performed, end-expiratory PCWP was used as a surrogate for LVEDP in the calculation of ΔP_TM_ when direct LVEDP measurements were unavailable. All pressure tracings were visually reviewed for physiological plausibility, and end-expiratory values were used for analysis.

### Statistical analysis

Continuous variables were summarized as mean ± standard deviation or median (interquartile range) and compared between groups using the independent samples *t*-test or Mann–Whitney *U* test, as appropriate. Categorical variables were expressed as counts and percentages and compared using the chi-square or Fisher’s exact test.

The primary outcome was ventilatory efficiency, assessed by the VE/VCO_2_ slope; peak VO_2_ was analysed as a secondary outcome. The main exposure was LV ΔP_TM_, defined as LVEDP—RAP, and analysed both as a continuous variable and by tertiles (T1 ≤9, T2 10–15, T3 ≥16).

Missing data were evaluated prior to analysis. Variables with >10% missingness for continuous variables or >5% for categorical variables were excluded and remaining missing values were imputed using missForest (excluding exposures and outcomes). Confounder selection was guided by a directed acyclic graph (DAG) based on physiological principles, yielding a minimally sufficient adjustment set, including age, sex, body mass index, LVEF, TAPSE, glomerular filtration rate, haemoglobin, chronic obstructive pulmonary disease, atrial fibrillation (AF), pulmonary vascular resistance, and ischaemic aetiology (Supplementary Figure S1).

Associations between ΔP_TM_ and CPET-derived outcomes were examined using multivariable Bayesian linear regression models. Models were constrained using regularizing, weakly informative prior distributions (β ∼ Normal (0,5) for adjustment covariates and β ∼ Normal (0,2) for the primary exposure). The model intercept and residual variance (σ) were specified using Student-t distributions. Detailed mathematical specifications of the prior frameworks, Markov Chain Monte Carlo sampling setups, and model calibration/convergence diagnostics are provided in the Supplementary Methods. Linearity assumptions were assessed using restricted cubic splines with 3 knots and linear specifications were retained. Model estimates are reported as posterior means with 95% credible intervals.

Formal model comparison was performed using leave-one-out cross-validation, the Widely Applicable Information Criterion (WAIC), and Bayes Factors to compare relative model fit and model parsimony across alternative parameterizations.

To assess the robustness of the findings, all primary Bayesian formulations were replicated and verified using traditional frequentist Ordinary Least Squares regression models using the identical covariate adjustment set (Supplementary Table S1). All analyses were performed in R version 4.4.0 using brms, tidybayes, posterior, missForest, rms, and ggplot2.

## Results

### Study population

A total of 534 patients were initially screened and 112 patients were excluded. The most common reason for exclusion was LVEF >25%, accounting for 63% of excluded patients (*n* = 71). Severe chronic pulmonary disease was present in 13% (*n* = 15), while incomplete or missing CPET or RHC data accounted for the remaining 24% (*n* = 26). Consequently, the final study population comprised 422 patients included in the analysis (Supplementary Figure S2).

### Baseline characteristics across transmural pressure tertiles

Descriptive clinical, echocardiographic, haemodynamic, laboratory, and CPET characteristics across ΔP_TM_ tertiles are summarized in *Table 1*. Demographic variables, including age and sex distribution, were comparable across groups.

**Table 1:** Clinical, echocardiographic, haemodynamic, laboratory, and CPET characteristics according to transmural pressure groups

Variable	T1 (*n* = 145)	T2 (*n* = 138)	T3 (*n* = 139)	*P*
***Clinical and demographic characteristics***				
Gender (female)	25 (17.2)	20 (14.5)	17 (12.2)	.490
Age (years)	51.1 (9.7)	51.7 (11.5)	51.11 (11.2)	.872
BMI (kg/m^2^)	28.1 (4.9)	28.3 (5.3)	27.1 (4.9)	.120
Ischaemic aetiology	56 (38.6)	77 (55.8)	65 (46.8)	.017
Hypertension	51 (35.2)	57 (41.6)	54 (38.8)	.537
Diabetes mellitus	46 (31.7)	59 (42.7)	42 (30.2)	.050
Hyperlipidaemia	49 (33.8)	60 (43.5)	51 (36.7)	.208
Smoker	104 (71.7)	102 (73.9)	95 (68.3)	.530
COPD	19 (13.1)	18 (13)	16 (11.5)	.896
CKD	30 (20.7)	34 (24.6)	22 (15.8)	.179
ICD	36 (24.8)	30 (21.7)	32 (23)	.841
CRT	10 (6.9)	10 (7.2)	9 (6.5)	.964
Atrial fibrillation	32 (22.1)	25 (18.1)	23 (16.5)	.477
***Echocardiographic parameters***				
LVEF (%)	22.0 (3.7)	21.3 (4.2)	21.2 (3.4)	.169
LVEDD (cm)	6.8 (0.9)	6.7 (0.7)	6.9 (0.8)	.448
LVESD (cm)	5.9 (1.0)	5.9 (0.7)	6.1 (0.9)	.463
LA diameter (AP, cm)	4.6 (0.6)	4.7 (0.5)	4.7 (0.5)	.096
MR grade^e^				.090
1	60 (42.3)	45 (33.1)	36 (27.1)	
2	52 (36.6)	65 (47.8)	65 (48.9)	
3	30 (21.1)	26 (19.1)	32 (24.1)	
TR grade				.048
1	82 (56.5)	70 (50.7)	71 (51.1)	
2	38 (26.2)	41 (29.7)	54 (38.8)	
3	25 (17.2)	27 (19.5)	13 (9.3)	
PASP (echo, mmHg)	35 (25–49.5)	40 (29.5–55)	45 (35–60)	<.001^a,b^
LV diastolic dysfunction grade^e^				<.001
1	35 (25.5)	25 (18.7)	11 (8.2)	
2	36 (26.3)	20 (14.9)	30 (22.4)	
3	66 (48.2)	89 (66.4)	93 (69.4)	
TAPSE (cm)	1.6 (0.4)	1.6 (0.5)	1.7 (0.4)	.039^c^
IVC diameter (cm)	1.9 (0.5)	2.0 (0.5)	2.0 (0.4)	.279
***Haemodynamic parameters (RHC)***				
Transmural pressure difference (mmHg)	6.0 (2.2)	12.6 (1.7)	20.6 (4)	<.001^d^
LVEDP (mmHg)	15.3 (6.7)	23.2 (5.8)	29.7 (6.1)	<.001^d^
RAP (mmHg)	7 (5–13)	10.0 (5.25–15)	8 (6–11)	.015^a^
RVSP (mmHg)	34.0 (28–47)	48.0 (38.3–62)	58.0 (50–68)	<.001^d^
PASP (mmHg)	33 (26–50)	50 (38–61.8)	59 (51–69.5)	<.001^d^
PADP (mmHg)	12 (9–24)	23.5 (16–29)	28 (22.5–33)	<.001^d^
PAMP (mmHg)	20 (15–33)	34 (26.3–41)	40 (34.5–47.5)	<.001^d^
TPG (mmHg)	7 (4–12)	10 (5.2–14)	9 (5–14)	.039^a^
TSG (mmHg)	75.5 (15.4)	79.2 (17.4)	79.2 (15.7)	.099
PVR (WU)	2.0 (1–3.8)	2.8 (1.5–4.5)	2.6 (1.5–4.3)	.008^a,b^
SV (ml)	46 (15.6)	40.6 (14.3)	41.5 (11.9)	.004^a,b^
SVI (ml/m^2^)	23.6 (7.9)	20.6 (6.8)	21.4 (6.2)	.006^a^
CO (L/min)	3.7 (1.1)	3.4 (0.9)	3.4 (0.8)	.054
CI (L/min/m^2^)	1.9 (0.5)	1.7 (0.4)	1.7 (0.4)	.065
SVR (WU)	30 (6.6)	23.5 (6.8)	23.8 (6.1)	.703
RVSWI (g·m/m^2^)	3.92 (2.83–5.44)	5.88 (4.34–7.50)	8.36 (6.36–10.30)	.001^d^
***Laboratory findings***				
Glucose (mg/dl)	109 (93.6–131)	112 (93.6–148)	111 (94.1–146)	.281
Urea (mg/dl)	42.3 (34.1–56)	46.1 (36.1–59.3)	41.4 (34–53.5)	.186
Creatinine (mg/dl)	1 (0.8–1.2)	1 (0.9–1.2)	1 (0.8–1.2)	.028^c^
AST (U/L)	21.6 (17–27.8)	21.2 (16–27.4)	20 (15.3–28.2)	.543
ALT (U/L)	21.6 (15.9–31.2)	21 (13.5–32.3)	21 (15–29.2)	.773
Total bilirubin (mg/dl)	0.8 (0.5–1.2)	0.8 (0.4–1.4)	0.7 (0.5–1.1)	.179
Direct bilirubin (mg/dl)	0.3 (0.2–0.6)	0.3 (0.2–0.5)	0.2 (0.2–0.5)	.127
ALP (U/L)	91 (72–118)	91 (73–128)	88 (67–112)	.461
GGT (U/L)	37 (25–72)	36 (21–73)	36 (19.5–84)	.751
NT-proBNP (pg/ml)	2273 (1154–4682)	2437 (1382–5305)	1834 (715–3865)	.024^c^
Triglycerides (mg/dl)	112 (88–167)	126 (88–186)	125 (90.7–171)	.554
LDL (mg/dl)	93.2 (65–127)	90.3 (66.5–115)	91.9 (62.6–122)	.872
HDL (mg/dl)	38 (31–46.9)	37.4 (31.7–46.7)	40 (33–50.6)	.173
Sodium (mmol/L)	138 (3.2)	138 (3.5)	138 (2.5)	.735
Potassium (mmol/L)	4.5 (0.5)	4.5 (0.5)	4.5 (0.5)	.527
Total protein (g/L)	70.5 (6.4)	71 (6.8)	70.8 (6.5)	.880
Albumin (g/L)	43.2 (4.4)	42.4 (5.6)	43 (5.1)	.505
LDH (U/L)	210 (184–264)	219 (190–276)	215 (183–265)	.377
eGFR (ml/min/1.73 m^2^)	82 (69–95)	80 (62–96.3)	85 (66.5–100)	.079
TSH (mIU/L)	1.9 (1.3–3)	1.9 (1.2–3)	1.9 (1.2–3)	.901
INR	1.2 (1.1–1.4)	1.2 (1.1–1.4)	1.1 (1.1–1.3)	.112
Haemoglobin (g/dl)	13.8 (1.9)	13.5 (2.1)	14 (2)	.085
Haematocrit (%)	43 (5.2)	42 (5.8)	43.4 (5.7)	.100
Platelet (10^3^/µl)	252 (207–288)	241 (197–314)	252 (215–292)	.865
***CPET parameters***				
Time (min)	6.5 (3.7–9)	6.2 (3.5–9)	7.4 (4.23–9.41)	.019^b,c^
Workload (watts)	80 (44.3–135)	80 (45–130)	108 (50.8–160)	.039^c^
V_E_ (L/min)	46.5 (38–53)	45 (38–54)	45 (38–54)	.996
VO_2_ (ml/min)	991 (720–1347)	1031 (765–1244)	1160 (844–1494)	.022^b,c^
Peak VO_2_ (ml/kg/min)	13 (10–16.2)	12.8 (10–15.7)	14.8 (11.4–17.6)	.005^b,c^
Predicted per cent (%)	28.2 (24.3–32)	27.5 (24.6–31.9)	29 (25.6–32.3)	.406
RER	1.04 (0.99–1.09)	1.03 (0.99–1.10)	1.01 (0.97–1.05)	<.001^b,c^
METs	3.7 (2.9–4.6)	3.6 (2.8–4.5)	4.2 (3.3–5)	.004b^c^
V_E_/VCO_2_ slope	40.2 (31.7–67.1)	39.5 (32.5–57.8)	34.9 (30.6–44.8)	.029b^c^
VO_2_/W slope [(ml/min)/W]	3.4 (1.9–4.2)	3.4 (1.9–5.4)	3.9 (2.2–5.6)	.417

Missing values were excluded from the percentage denominators as follows: MR grade was missing in 11 patients (T1: *n* = 3, T2: *n* = 2, T3: *n* = 6), and LV diastolic dysfunction grade was missing in 17 patients (T1: *n* = 8, T2: *n* = 4, T3: *n* = 5).

AF, atrial fibrillation; ALP, alkaline phosphatase; ALT, alanine aminotransferase; AP, anteroposterior; AST, aspartate aminotransferase; BMI, body mass index; CI, cardiac index; CKD, chronic kidney disease; CO, cardiac output; COPD, chronic obstructive pulmonary disease; CPET, cardiopulmonary exercise testing; CRT, cardiac resynchronization therapy; eGFR, estimated glomerular filtration rate; GGT, gamma-glutamyl transferase; HDL, high-density lipoprotein cholesterol; ICD, implantable cardioverter-defibrillator; INR, international normalized ratio; IVC, inferior vena cava; LA, left atrium; LDH, lactate dehydrogenase; LDL, low-density lipoprotein cholesterol; LV, left ventricle; LVAD, left ventricular assist device; LVEDD, left ventricular end-diastolic diameter; LVEDP, left ventricular end-diastolic pressure; LVEF, left ventricular ejection fraction; LVESD, left ventricular end-systolic diameter; METs, metabolic equivalents; MR, mitral regurgitation; NT-proBNP, N-terminal pro-B-type natriuretic peptide; PADP, pulmonary artery diastolic pressure; PAMP, mean pulmonary artery pressure; PASP, pulmonary artery systolic pressure; PCWP, Pulmonary capillary wedge pressure; RAP, right atrial pressure; RER, respiratory exchange ratio; RHC, right heart catheterization; RV, right ventricle; RVSP, right ventricular systolic pressure; RVSWI, right ventricular stroke work index; SVI, stroke volume index; SVR, systemic vascular resistance; TAPSE, tricuspid annular plane systolic excursion; TPG, transpulmonary gradient; TR, tricuspid regurgitation; TSG, transsystemic gradient; VCO_2_, carbon dioxide production; VE/VCO_2_, carbon dioxide ventilatory equivalent; VE, ventilation; VO2/W, oxygen consumption/workload slope; VO_2_, oxygen consumption; W, watts.

^a^T1 vs T2.

^b^T1 vs T3.

^c^T2 vs T3.

^d^All pairwise comparisons significant.

^e^Percentages for mitral regurgitation severity and LV diastolic dysfunction grade are calculated based on complete cases with evaluable data within each tertile.

The prevalence of ischaemic aetiology differed significantly among ΔP_TM_ tertiles, whereas conventional cardiovascular risk factors, comorbidities, and device therapy were comparable.

Echocardiographically, LVEF, ventricular dimensions, and other measured parameters were similar across tertiles. In contrast, tricuspid regurgitation grade, pulmonary artery systolic pressure, LV diastolic dysfunction grade, and TAPSE differed significantly among tertiles.

Invasive haemodynamic parameters showed marked differences across ΔP_TM_ strata. Higher ΔP_TM_ was associated with higher pulmonary artery pressures. Transpulmonary gradient, pulmonary vascular resistance, stroke volume, stroke volume index, and right ventricular stroke work index differed significantly among groups, whereas cardiac output, cardiac index, and systemic vascular resistance were comparable (*Figure 1*).

**Figure 1:**
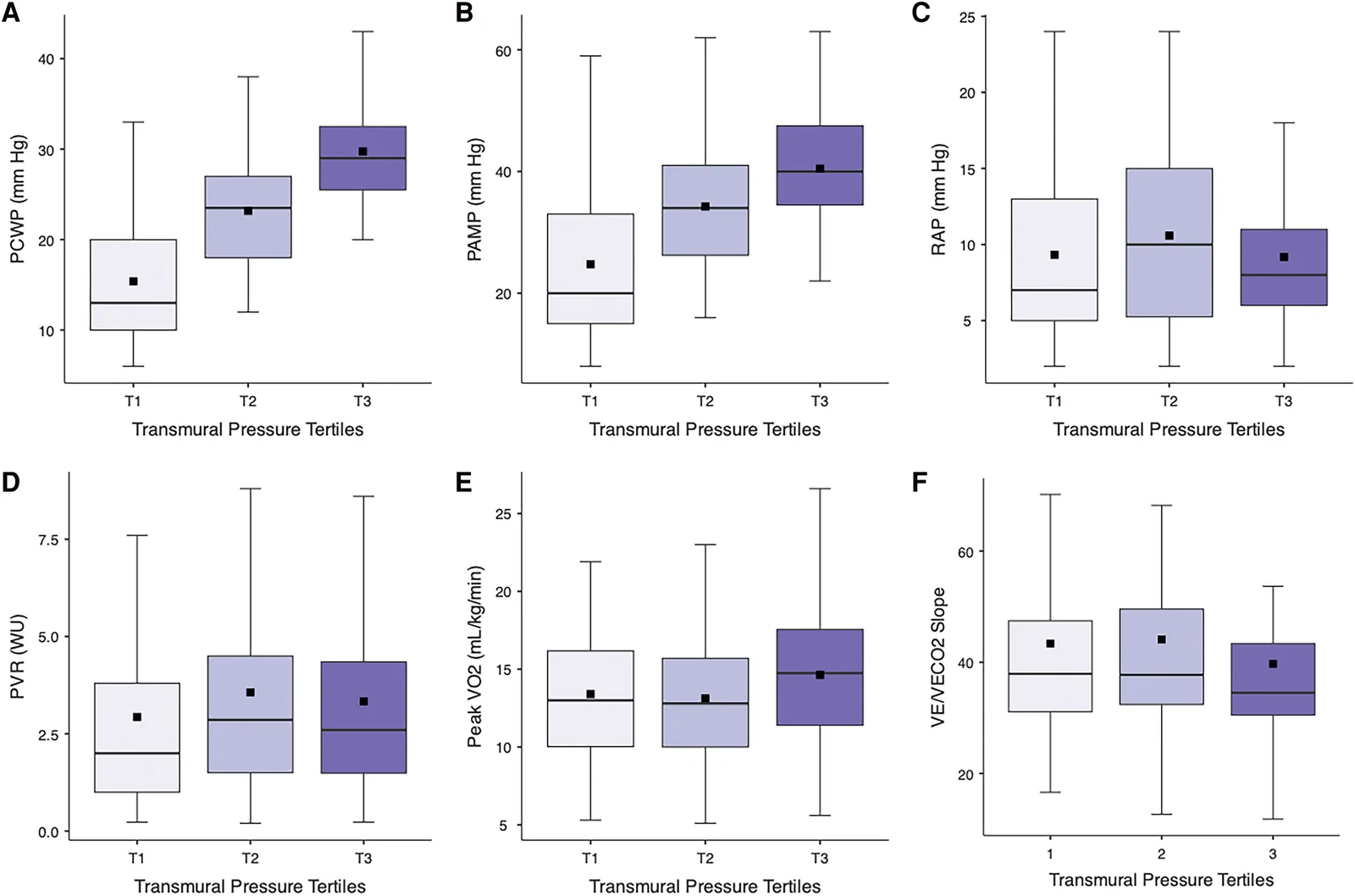
Haemodynamic and cardiopulmonary exercise parameters across transmural pressure difference (ΔP_TM_) tertiles. Boxplots depict comparisons across ΔP_TM_ tertiles (T1 ≤9, T2 10–15, T3 ≥16 mmHg) for (*A*) left ventricular end-diastolic pressure (LVEDP), (*B*) mean pulmonary artery pressure (PAMP), (*C*) right atrial pressure (RAP), (*D*) pulmonary vascular resistance, (*E*) peak oxygen uptake (peak VO_2_), and (*F*) ventilatory efficiency assessed by the V_E_/VCO_2_ slope

Laboratory parameters were largely comparable across ΔP_TM_ groups; however, creatinine and N-terminal pro-brain natriuretic peptide levels differed significantly among tertiles. All other hepatic, electrolyte, and lipid parameters were similar between groups.

CPET revealed significant differences in exercise time, peak oxygen uptake indexed to body weight, respiratory exchange ratio, and V_E_/VCO_2_ slope across ΔP_TM_ tertiles (*Figure 1*). Absolute peak VO_2_ also differed across ΔP_TM_ tertiles, whereas predicted peak VO_2_% and VO_2_/work slope did not differ significantly among groups.

### Comparison of ΔP_TM_ and ventilatory efficiency

In multivariable Bayesian regression models adjusted for prespecified confounders, higher ΔP_TM_ was associated with a lower VE/VCO_2_ slope, consistent with better ventilatory efficiency (*Table 2*). The posterior mean slope for ΔP_TM_ was −0.34 (95% credible interval [CrI] −0.58 to −0.11), corresponding to a 99.7% posterior probability of a negative association. Across tertiles, patients in the highest ΔP_TM_ group (T3) had lower V_E_/VCO_2_ slopes than those in the lowest tertile (T1), with an adjusted posterior predictive median difference of −4.42 units (95% CrI −7.85 to −0.62) and a low posterior probability of a positive difference (*P* [Δ>0] = .005). No evidence of nonlinearity was observed. The posterior mean relationship is illustrated in *Figures 2* and *3*.

**Figure 2:**
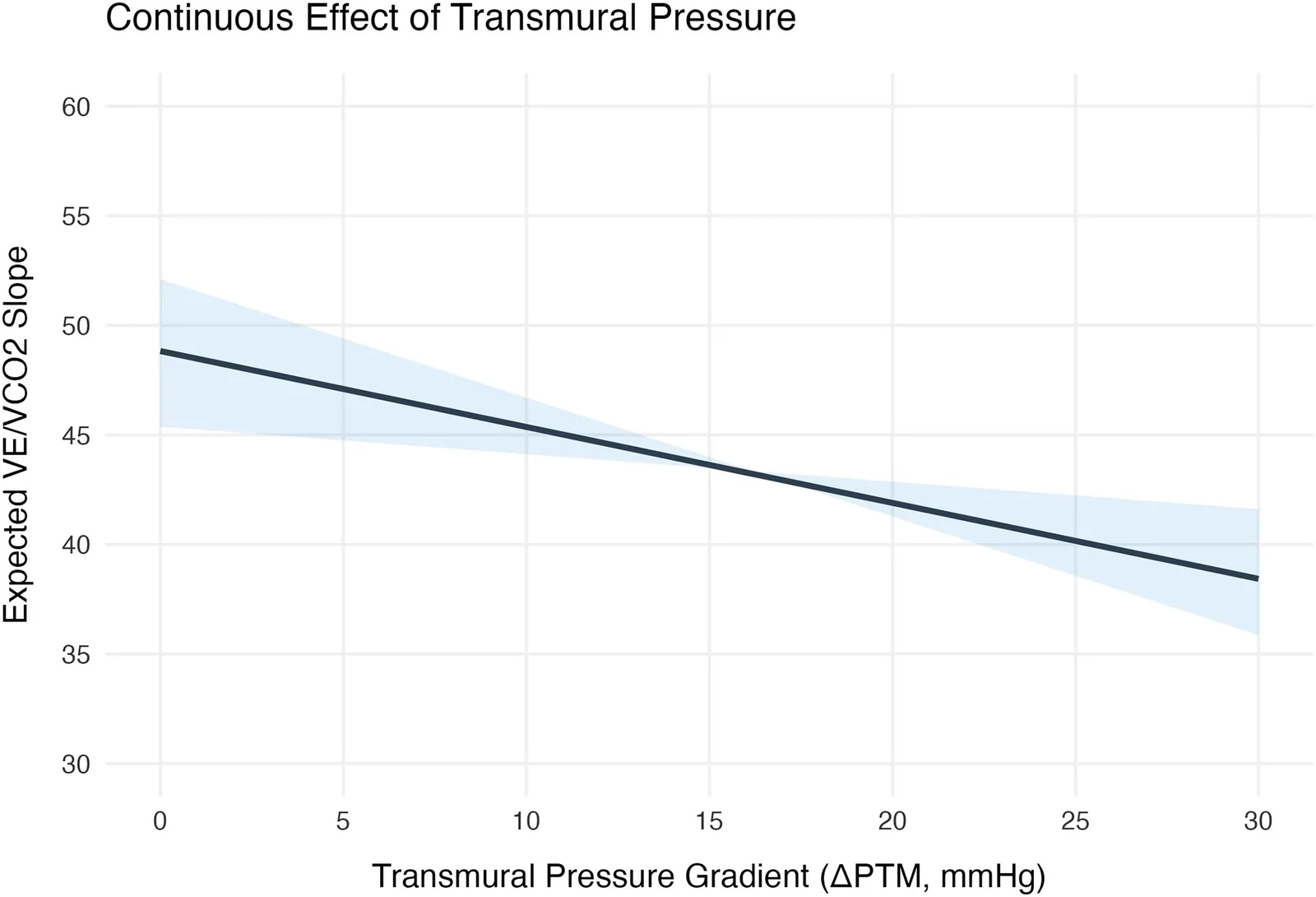
Continuous association between rested transmural pressure gradient and ventilatory efficiency. Multivariable Bayesian regression conditional effects plot demonstrating the continuous relationship between the resting left ventricular transmural pressure gradient and the expected V_E_/VCO_2_ slope during maximal exertion. The solid dark line represents the estimated posterior mean trajectory. The light-blue shaded area represents the corresponding 95% Bayesian credible interval. The stable downward trajectory highlights that higher ΔP_TM_ values were associated with lower V_E_/VCO_2_ slopes

**Figure 3:**
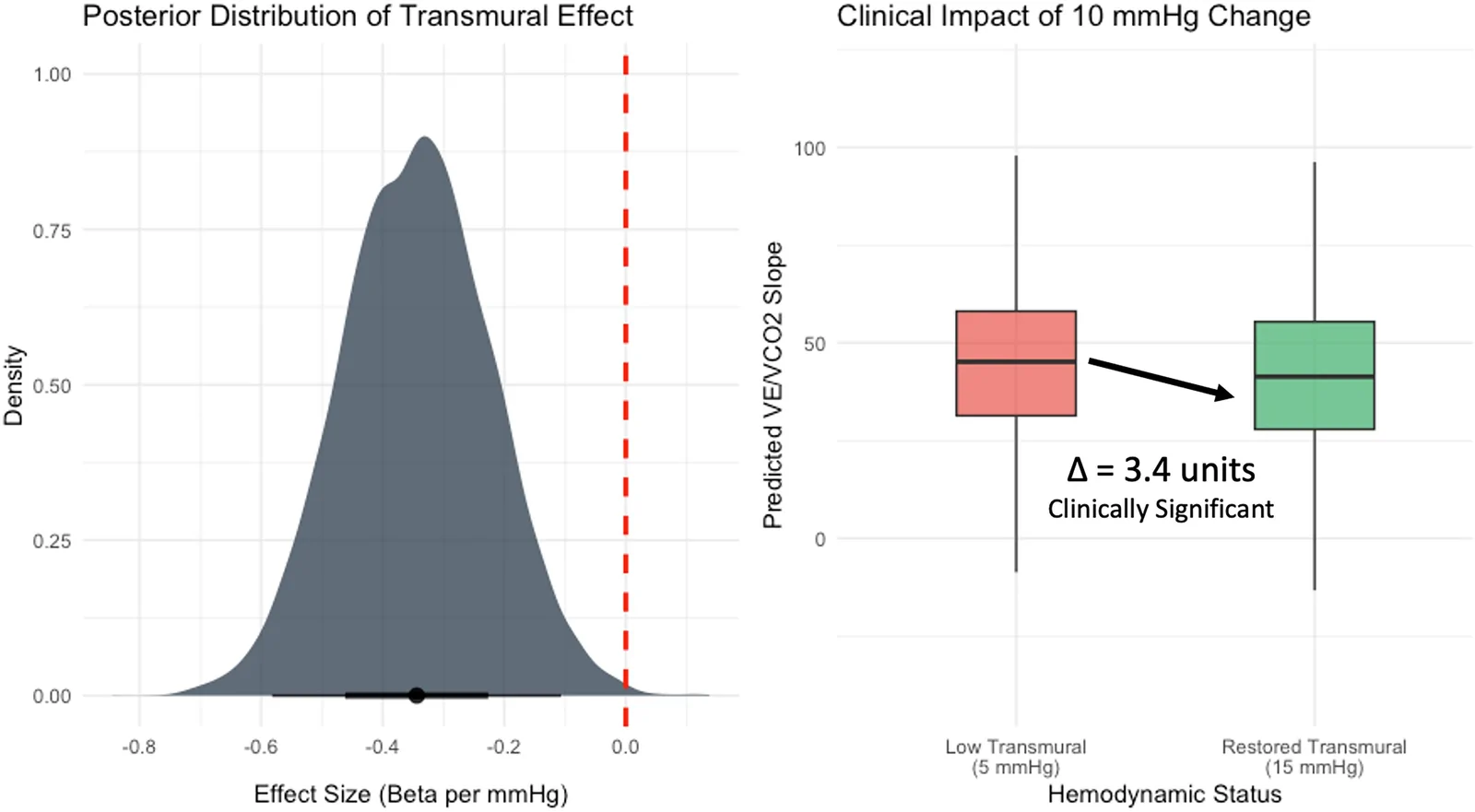
Bayesian posterior density and clinical impact of transmural pressure verification. (Left panel) Posterior probability density distribution for the primary exposure coefficient (β per mmHg change in ΔPTM). The central black point and the solid horizontal bar denote the posterior mean (β = −0.34) and the 95% Bayesian credible interval (−0.58 to −0.11), respectively. The vertical red dashed line indicates the null value, which is excluded by the posterior distribution, corresponding to a 99.7% posterior probability of a negative association. (Right panel) Posterior predictive contrasts illustrating the model-estimated effect of a hypothetical 10-mmHg increase in ΔPTM. A hypothetical increase in ΔPTM from 5 mmHg (orange box) to 15 mmHg (green box) corresponds to an expected mean reduction of 3.4 units in the VE/VCO_2_ slope (Δ = −3.4), consistent with better ventilatory efficiency

**Table 2:** Multivariable Bayesian associations between left ventricular transmural pressure and ventilatory efficiency (V_E_/VCO_2_ slope)

Predictor	Posterior mean (β)	Est. error	95% credible interval (CrI)
Transmural pressure difference (mmHg)	−0.34	0.12	−0.58 to −0.11
Atrial fibrillation	2.40	1.29	−0.15 to 4.95
Pulmonary vascular resistance (wood units)	−0.21	0.40	−0.97 to 0.57
Gender (female)	−0.27	1.35	−2.92 to 2.36
Age (years)	0.05	0.10	−0.14 to 0.25
BMI (kg/m^2^)	0.08	0.21	−0.33 to 0.48
LVEF (%)	−0.16	0.28	−0.71 to 0.40
TAPSE (cm)	0.16	1.28	−2.29 to 2.69
eGFR (ml/min/1.73 m^2^)	−0.12	0.05	−0.21 to −0.03
Haemoglobin (g/dl)	−2.42	0.51	−3.42 to −1.43
COPD	0.65	1.35	−1.97 to 3.27
Ischaemic aetiology	−0.56	1.25	−2.99 to 1.93

BMI, body mass index; COPD, chronic obstructive pulmonary disease; CrI, credible interval; eGFR, estimated glomerular filtration rate; LVEF, left ventricular ejection fraction; TAPSE, tricuspid annular plane systolic excursion; VE/VCO_2_, carbon dioxide ventilatory equivalent.

### Comparison of ΔP_TM_ with filling pressures

When ΔP_TM_ was replaced by its individual haemodynamic components, the associations with ventilatory efficiency were substantially attenuated or characterized by high uncertainty. Substituting ΔP_TM_ with LVEDP alone resulted in a weaker association with the VE/VCO_2_ slope that crossed the null interval (posterior mean −0.25; 95% CrI, −0.55 to 0.04), indicating limited explanatory capacity of LVEDP as an isolated haemodynamic marker (*Table 3*, Model A). Similarly, RAP modelled individually demonstrated no robust association with ventilatory efficiency (posterior mean −0.24; 95% CrI, −0.62 to 0.14; *Table 3*, Model B).

**Table 3:** Multivariable Bayesian associations between alternative haemodynamic variables and ventilatory efficiency (V_E_/VCO_2_ slope)

Predictor	Model 1: transmural (ΔP_TM_)	Model A: LVEDP	Model B: RAP	Model C: joint (LVEDP + RAP)
**Primary haemodynamic exposure**				
Transmural pressure (mmHg)	−0.34 (−0.58 to −0.11)	—	—	—
LVEDP (mmHg)	—	−0.25 (−0.55 to 0.04)	—	−0.25 (−0.66 to 0.16)
RAP (mmHg)	—	—	−0.24 (−0.62 to 0.14)	−0.01 (−0.55 to 0.53)
**Adjustment set**				
Atrial fibrillation	2.40 (−0.15 to 4.95)	2.45 (−0.17 to 5.10)	2.41 (−0.20 to 5.01)	2.42 (−0.17 to 5.02)
PVR (wood units)	−0.21 (−0.97 to 0.57)	−0.05 (−0.87 to 0.77)	−0.13 (−0.95 to 0.68)	−0.05 (−0.86 to 0.78)
Sex (female)	−0.27 (−2.92 to 2.36)	−0.29 (−2.94 to 2.34)	−0.37 (−2.99 to 2.29)	−0.31 (−2.85 to 2.30)
Age (years)	0.05 (−0.14 to 0.25)	0.03 (−0.17 to 0.23)	0.03 (−0.16 to 0.23)	0.03 (−0.17 to 0.24)
BMI (kg/m^2^)	0.08 (−0.33 to 0.48)	0.12 (−0.29 to 0.53)	0.14 (−0.27 to 0.55)	0.12 (−0.29 to 0.54)
LVEF (%)	−0.16 (−0.71 to 0.40)	−0.19 (−0.75 to 0.37)	−0.16 (−0.72 to 0.40)	−0.19 (−0.76 to 0.37)
TAPSE (cm)	0.16 (−2.29 to 2.69)	−0.08 (−2.58 to 2.41)	−0.11 (−2.61 to 2.40)	−0.09 (−2.68 to 2.53)
eGFR (ml/min/1.73\m^2^)	−0.12 (−0.21 to −0.03)	−0.12 (−0.21 to −0.03)	−0.11 (−0.20 to −0.02)	−0.12 (−0.21 to −0.03)
Haemoglobin (g/dL)	−2.42 (−3.42 to −1.43)	−2.42 (−3.41 to −1.42)	−2.40 (−3.39 to −1.40)	−2.42 (−3.39 to −1.43)
COPD	0.65 (−1.97 to 3.27)	0.72 (−1.94 to 3.41)	0.73 (−1.91 to 3.37)	0.70 (−1.94 to 3.37)
Ischaemic aetiology	−0.56 (−2.99 to 1.93)	−0.48 (−2.88 to 2.01)	−0.48 (−2.91 to 2.02)	−0.46 (−2.89 to 2.01)
**Bayesian model fit and comparison**				
Residual SD (σ)	19.05 (17.67 to 20.55)	19.01 (17.60 to 20.52)	19.07 (17.67 to 20.57)	19.04 (17.64 to 20.59)
Expected parameter penalty	9.2	9.8	9.7	10.7
WAIC metric	2986.6	2986.0	2987.3	2987.9
LOO-CV comparison (ΔELPD)	−0.3 (SE = 1.4)	0.0 (Reference)	−0.6 (SE = 1.2)	−0.9 (SE = 0.1)
Bayes factor (BF_TM vs._)	1.0 (Reference)	2.98	4.65	16.10

BMI, body mass index; COPD, chronic obstructive pulmonary disease; CrI, credible interval; eGFR, estimated glomerular filtration rate; LOO-CV, Leave-one-out cross-validation; LVEDP, Left ventricle end diastolic pressure Pulmonary capillary wedge pressure; LVEF, left ventricular ejection fraction; PVR, pulmonary vascular resistance; RAP, right atrial pressure; TAPSE, tricuspid annular plane systolic excursion; VE/VCO_2_, carbon dioxide ventilatory equivalent; WAIC, Watanabe-Akaike information criterion.

When LVEDP and RAP were entered simultaneously into the same model, neither component demonstrated a robust independent association with VE/VCO_2_ slope (LVEDP: posterior mean −0.25, 95% CrI −0.66 to 0.16; RAP: posterior mean −0.01, 95% CrI −0.55 to 0.53; *Table 3*, Model C). Formal Bayesian model comparisons demonstrated comparable predictive performance across all model specifications, with minimal differences in WAIC and leave-one-out cross-validation metrics. Accordingly, the principal advantage of the ΔP_TM_ formulation lies in its greater interpretive parsimony rather than superior predictive performance.

### Comparison of ΔP_TM_ and peak VO_2_

In exploratory multivariable analyses adjusted for the full confounding set, neither ΔP_TM_ nor conventional filling pressures showed a meaningful or statistically stable association with maximal exercise capacity. For the primary exposure, the posterior mean slope of ΔP_TM_ predicting peak VO_2_ was +0.06 (95% CrI, −0.02 to +0.14). Conversely, posterior distributions for LVEDP and RAP, modelled individually or jointly, were centred around the null value, indicating no clinically meaningful association with peak aerobic capacity (Supplementary Tables S2 and S3).

## Discussion

In this cohort of patients with advanced systolic HF undergoing invasive haemodynamic assessment and CPET, resting LV transmural pressure difference was inversely associated with ventilatory inefficiency, as reflected by the V_E_/VCO_2_ slope. This association remained evident after multivariable adjustment, whereas LVEDP and RAP considered individually did not demonstrate robust independent associations with ventilatory efficiency. In contrast, ΔP_TM_ was not associated with peak VO_2_. These findings suggest that the balance between left-sided filling pressure and right-sided/pericardial constraint may be related to exercise ventilatory efficiency in advanced HF. A posterior mean β coefficient of −0.34 implies that for every 10-mmHg reduction in resting ΔP_TM_, the V_E_/VCO_2_ slope increases by approximately 3.4 units. Given that the V_E_/VCO_2_ slope is a continuous marker of gas exchange efficiency and ventilation–perfusion matching, this sub-clinical shift underscores how even modest changes in the resting balance between intracavitary filling and external constraint manifest as measurable ventilatory inefficiency during exercise. The observed weak associations between resting LVEDP, RAP, and exercise-related measures, such as V_E_/VCO_2_ slope and peak VO_2_, are consistent with prior invasive exercise haemodynamic studies.^6,7,17,18^ Patients with normal resting filling pressures may develop marked haemodynamic abnormalities during exercise, indicating that functional limitation is driven primarily by dynamic rather than static responses.^6,7^ On the other hand, ΔP_TM_ offers a different way to summarize resting haemodynamics by incorporating both LV filling pressure and the opposing external constraint approximated by RAP. Smiseth *et al*.^6^ demonstrated that pericardial pressure closely approximates RAP, making RAP correction physiologically necessary to estimate true LV distending pressure. Clinically, this is evident in conditions where elevated right-sided pressures cause LVEDP to overestimate effective LV diastolic distension. This principle is supported by Aslanger et al.^7^ showing that elevated PCWP does not necessarily reflect increased effective preload, particularly in phenotypes with high RAP, where true transmural distension may be masked by elevated intracavitary pressures. Heart-lung interaction studies further reinforce this framework. Leahy et al.^19^ demonstrated that in HF with preserved LVEF, dynamic hyperinflation and increased intrathoracic pressure during exercise are associated with higher measured PCWP, suggesting that exercise-induced PCWP elevation may partly reflect respiratory mechanics rather than intrinsic ventricular stiffness alone. These observations underscore the limitations of interpreting intracavitary pressures without accounting for pericardial and respiratory constraints.

In unadjusted analyses, patients in the highest ΔP_TM_ tertile exhibited better baseline functional parameters, including longer exercise duration, higher peak VO_2_, lower V_E_/VCO_2_ slope, and lower NT-proBNP levels despite elevated LVEDP and pulmonary pressures. This pattern demonstrates that ΔP_TM_ does not simply reflect global volume overload or advanced disease severity. Instead, a higher resting ΔP_TM_ marks a haemodynamic phenotype defined by left-sided distending pressure exceeding right-sided constraint.

Although unadjusted peak VO_2_ differed across tertiles, this relationship was eliminated after multivariable adjustment. Conversely, the association between ΔP_TM_ and V_E_/VCO_2_ slope persisted. The baseline functional differences observed across tertiles highlight potential unmeasured phenotypic heterogeneity. Accordingly, these observational data indicate that ΔP_TM_ identifies a specific haemodynamic profile rather than demonstrating a direct causal effect on exercise capacity.

Within this context, our findings suggest that ΔP_TM_ may provide a more integrated summary of resting haemodynamic conditions than either filling pressure considered in isolation. Although LVEDP and RAP represent distinct physiological processes, neither demonstrated a robust independent association with V_E_/VCO_2_ slope when modelled individually or jointly. In contrast, ΔP_TM_ maintained a consistent inverse association with ventilatory inefficiency across the primary analyses. This pattern should not be interpreted as evidence that LVEDP and RAP lack independent physiological importance. Rather, it suggests that, within this cohort, the individual contributions of these closely related pressure measurements could not be reliably distinguished, whereas their composite parameterization may more parsimoniously summarize the balance between left-sided filling pressure and right-sided/pericardial constraint.

Because ΔP_TM_ is mathematically derived from LVEDP and RAP, comparisons between the composite index and its component pressures should be interpreted cautiously. Formal Bayesian model comparisons demonstrated comparable predictive performance across competing model specifications. Accordingly, the principal value of the ΔP_TM_ framework lies in providing a physiologically interpretable and parsimonious summary of correlated haemodynamic information rather than in demonstrating superior predictive performance or establishing a unique physiological mechanism. ΔP_TM_ should therefore be regarded as a composite haemodynamic parameter reflecting the balance between left-sided filling pressure and right-sided/pericardial constraint rather than as a uniquely identifiable physiological construct. The present data do not permit attribution of the observed association to either LVEDP or RAP individually, nor do they allow discrimination among alternative physiological mechanisms underlying the composite ΔP_TM_ measure. Future studies incorporating simultaneous invasive exercise haemodynamics and external validation will be required to clarify the relative physiological contributions of the individual pressure components.

The present study does not establish ΔP_TM_ as a therapeutic target or as a substitute for exercise haemodynamic testing. A higher ΔP_TM_ may arise from several non-mutually exclusive mechanisms, including greater left-sided filling, less opposing right-sided/pericardial constraint, more favourable RV-pulmonary vascular coupling, or reduced LV compliance. These possibilities have different physiological and clinical implications. The present data cannot distinguish among them. Accordingly, the present findings should be interpreted as identifying a resting haemodynamic phenotype associated with ventilatory efficiency rather than demonstrating a direct mechanistic role of ΔP_TM_ in exercise performance.

The association with V_E_/VCO_2_ slope but not peak VO_2_ is noteworthy and highlights the distinct physiological constructs these metrics represent. Peak VO_2_ is a highly multifactorial, composite measure that integrates not only central cardiac output but also a wide array of extracardiac and peripheral determinants. In advanced HF, peak aerobic capacity is frequently constrained by non-haemodynamic boundary conditions, including concomitant anaemia, intrinsic pulmonary ventilation defects, skeletal muscle deconditioning or wasting, and chronotropic incompetence. These peripheral and systemic factors effectively decouple maximal oxygen uptake from resting central filling profiles.^20^ By contrast, V_E_/VCO_2_ slope is closely related to ventilation-perfusion matching, physiological dead space, pulmonary vascular function, ventilatory control, and circulatory response during exertion.^21,22^ These processes may be more sensitive to the combined influence of left-sided filling conditions, right-sided loading, and pulmonary vascular coupling.^12,23,24^ This may explain why ΔP_TM_ showed a clearer relationship with ventilatory efficiency than with peak aerobic capacity.

Several clinical implications may be considered. First, the findings support the concept that left-sided filling pressure alone may be insufficient to characterize the haemodynamic substrate of ventilatory inefficiency in advanced HF. Second, incorporating right-sided pressure into composite haemodynamic indices may facilitate characterization of resting loading conditions among patients with similar LVEDP. Third, ΔP_TM_ may serve as a useful exploratory variable in future studies integrating resting catheterization, exercise haemodynamics, echocardiographic measures of ventricular interaction, and clinical outcomes.

### Limitations

Several limitations should be acknowledged. First, the cohort was derived exclusively from a highly selected advanced HF and heart transplantation evaluation clinic. This strict enrolment criterion introduces a substantial risk of selection bias and significantly limits the generalizability of our findings to broader, less severe HF populations. The observed relationships regarding transmural haemodynamics may strictly apply to this end-stage phenotype, where RV failure, secondary pulmonary hypertension, and pericardial constraint are advanced, and therefore should not be extrapolated to the general HF population.

Second, RHC, CPET, and TTE were not performed simultaneously; although obtained within a narrow interval (median: 3 days; maximum: 1 week), temporal variation in volume status, short-term medical therapy adjustments, and clinical stability may have introduced measurement variability and potential misclassification, thereby attenuating the underlying physiological relationship between resting transmural haemodynamics and exercise performance. Furthermore, because invasive haemodynamics were not assessed during exercise, the proposed downstream pathway linking impaired resting ΔP_TM_ to reduced exercise stroke volume augmentation and secondary pulmonary congestion remains hypothesis-generating.

Third, ΔP_TM_ was estimated as LVEDP minus RAP, based on the physiological assumption that RAP approximates pericardial pressure. Although supported by experimental and intraoperative observations suggesting that RAP tracks pericardial pressure changes and provides a clinically useful estimate of transmural filling pressure,^3^ this assumption has not been fully validated across all HF phenotypes and loading conditions and may vary according to right ventricular dilation, pericardial restraint, and intrathoracic pressure dynamics.^5^

Fourth, measurement error in PCWP remains a relevant concern, particularly in advanced HF, where large V-waves, respiratory variation, catheter position, and wedge quality may affect accuracy. Although all pressure tracings were visually reviewed and end-expiratory values were used for analysis, residual misclassification cannot be excluded. In addition, cardiac output was estimated using the indirect Fick method, which may introduce error when assumptions regarding oxygen consumption are violated.

Fifth, because ΔP_TM_ is mathematically derived from LVEDP and RAP, it is not separately identifiable from its constituent pressure components within the present observational framework. Consequently, the independent physiological contributions of LVEDP and RAP cannot be disentangled, and the present findings should not be interpreted as establishing an independent causal effect or unique physiological role of transmural pressure itself.

Sixth, ventilatory inefficiency is influenced by multiple factors beyond central haemodynamics, including pulmonary vascular disease, ventilatory control, anaemia, skeletal muscle conditioning, and treatment effects. Although adjustment was guided by a DAG-derived minimally sufficient confounder set and supported by sensitivity analyses, residual confounding cannot be completely excluded. All patients were receiving optimized medical therapy as part of advanced HF and heart transplantation care; however, detailed information on individual drug classes, doses, and treatment intensity was not available for the present analysis. Accordingly, potential confounding related to differences in pharmacological treatment intensity could not be specifically evaluated.

Finally, the absence of longitudinal follow-up data on hard clinical outcomes, including mortality, heart failure hospitalization, and transplantation, limits the prognostic implications of the present findings. Accordingly, this study should be regarded as primarily observational and cross-sectional, and the findings should be considered hypothesis-generating rather than prognostic.

## Conclusion

This study showed that LV ΔP_TM_ was inversely associated with V_E_/VCO_2_ slope in patients with advanced systolic HF, whereas its association with peak VO_2_ was minimal. These findings suggest that ΔP_TM_ may serve as a parsimonious composite haemodynamic parameter summarizing the balance between left-sided filling pressure and right-sided/pericardial constraint associated with ventilatory efficiency. Further prospective studies incorporating simultaneous exercise haemodynamics and clinical outcomes are warranted to validate these findings.

## Supplementary Material

xvag247_Supplementary_Data

## Declarations

### Data Availability

No data were generated or analysed for this manuscript.

### Pre-registered Clinical Trial Number

None supplied.

## References

[1] Borlaug BA, Nishimura RA, Sorajja P, Lam CSP, Redfield MM. Exercise hemodynamics enhance diagnosis of early heart failure with preserved ejection fraction. Circ Heart Fail 2010;3:588–95. 10.1161/CIRCHEARTFAILURE.109.930701 PMC3048586 20543134

[2] Eisman AS, Shah RV, Dhakal BP, Pappagianopoulos PP, Wooster L, Bailey C, et al Pulmonary capillary wedge pressure patterns during exercise predict exercise capacity and incident heart failure. Circ Heart Fail 2018;11:e004750. 10.1161/CIRCHEARTFAILURE.117.004750 PMC5937988 29695381

[3] Smiseth OA, Refsum H, Tyberg JV. Pericardial pressure assessed by right atrial pressure: a basis for calculation of left ventricular transmural pressure. Am Heart J 1984;108:603–5. 10.1016/0002-8703(84)90430-7 6433685

[4] Traboulsi M, Scott-Douglas NW, Smith ER, Tyberg JV. The right and left ventricular intracavitary and transmural pressure-strain relationships. Am Heart J 1992;123:1279–87. 10.1016/0002-8703(92)91034-X 1575146

[5] Tyberg JV, Taichman GC, Smith ER, Douglas NW, Smiseth OA, Keon WJ. The relationship between pericardial pressure and right atrial pressure: an intraoperative study. Circulation 1986;73:428–32. 10.1161/01.CIR.73.3.428 3948353

[6] Smiseth OA, Thompson CR, Ling H, Robinson M, Miyagishima RT. A potential clinical method for calculating transmural left ventricular filling pressure during positive end-expiratory pressure ventilation: an intraoperative study in humans. J Am Coll Cardiol 1996;27:155–60. 10.1016/0735-1097(95)00420-3 8522690

[7] Aslanger E, Akaslan D, Ataş H, Yıldırımtürk Ö, Öz M, Kocakaya D, et al Is pulmonary capillary wedge pressure a reliable indicator of postcapillary pulmonary hypertension? Am J Cardiol 2024;211:307–15. 10.1016/j.amjcard.2023.11.034 37984643

[8] Mancini DM, Eisen H, Kussmaul W, Mull R, Edmunds LH, Wilson JR. Value of peak exercise oxygen consumption for optimal timing of cardiac transplantation in ambulatory patients with heart failure. Circulation 1991;83:778–86. 10.1161/01.CIR.83.3.778 1999029

[9] Humbert M, Kovacs G, Hoeper MM, Badagliacca R, Berger RMF, Brida M, et al 2022 ESC/ERS guidelines for the diagnosis and treatment of pulmonary hypertension. Eur Respir J 2023;61:2200879. 10.1183/13993003.00879-2022 36028254

[10] Deboeck G, Scoditti C, Huez S, Vachiéry JL, Lamotte M, Sharples L, et al Exercise testing to predict outcome in idiopathic *versus* associated pulmonary arterial hypertension. Eur Respir J 2012;40:1410–9. 10.1183/09031936.00217911 22441747

[11] Arena R, Humphrey R, Peberdy MA. Prognostic ability of VE/VCO 2 slope calculations using different exercise test time intervals in subjects with heart failure. Eur J Cardiovasc Prev Rehabil 2003;10:463–8. 10.1097/01.hjr.0000102817.74402.5b 14671470

[12] Guazzi M, Arena R, Ascione A, Piepoli M, Guazzi MD. Exercise oscillatory breathing and increased ventilation to carbon dioxide production slope in heart failure: an unfavorable combination with high prognostic value. Am Heart J 2007;153:859–67. 10.1016/j.ahj.2007.02.034 17452165

[13] Poggio R, Arazi HC, Giorgi M, Miriuka SG. Prediction of severe cardiovascular events by VE/VCO_2_ slope versus peak Vo2 in systolic heart failure: a meta-analysis of the published literature. Am Heart J 2010;160:1004–14. 10.1016/j.ahj.2010.08.037 21146651

[14] Goncalves AV, Pereira-Da-Silva T, Soares R, Feliciano J, Abreu A, Rio P, et al Prognostic value of VE/VCO 2 slope in overweight heart failure patients. Am J Cardiovasc Dis 2020;20:578–84. PMC7811917 33489461

[15] Rudski LG, Lai WW, Afilalo J, Hua L, Handschumacher MD, Chandrasekaran K, et al Guidelines for the echocardiographic assessment of the right heart in adults: a report from the American Society of Echocardiography. Endorsed by the European Association of Echocardiography, a registered branch of the European Society of Cardiology, and the Canadian Society of Echocardiography. J Am Soc Echocardiogr 2010;23:685–788. 10.1016/j.echo.2010.05.010 20620859

[16] Mukherjee M, Rudski LG, Addetia K, Afilalo J, D’Alto M, Freed BH, et al Guidelines for the echocardiographic assessment of the right heart in adults and special considerations in pulmonary hypertension: recommendations from the American Society of Echocardiography. J Am Soc Echocardiogr 2025;38:141–86. 10.1016/j.echo.2025.01.006 40044341

[17] Hortegal R, Reddy YNV, Jain CC, Borlaug BA, Tedford R, Miranda WR. Agreement between PAWP and LVEDP during invasive cardiopulmonary exercise testing and comparison of prognostic performances. J Card Fail 2026;32:1323–32. 10.1016/j.cardfail.2025.10.017 41213319

[18] Jain CC, Borlaug BA. Performance and interpretation of invasive hemodynamic exercise testing. Chest 2020;158:2119–29. 10.1016/j.chest.2020.05.552 PMC7674988 32473950

[19] Leahy MG, Wakeham DJ, MacNamara JP, Brazile T, Abulimiti A, Hearon CM, et al Heart-lung interactions in HFpEF: dynamic hyperinflation and exercise PCWP. JACC Heart Fail 2025;13:102523. 10.1016/j.jchf.2025.102523 PMC12328072 40578265

[20] Task Force of the Italian Working Group on Cardiac Rehabilitation Prevention; Working Group on Cardiac Rehabilitation and Exercise Physiology of the European Society of Cardiology; Piepoli MF, Corrà U, Agostoni PG, Belardinelli R, et al Statement on cardiopulmonary exercise testing in chronic heart failure due to left ventricular dysfunction: recommendations for performance and interpretation. Part I: definition of cardiopulmonary exercise testing parameters for appropriate use in chronic heart failure. Eur J Cardiovasc Prev Rehabil 2006;13:150–64. 10.1097/01.hjr.0000209812.05573.04 16575267

[21] Toste A, Soares R, Feliciano J, Andreozzi V, Silva S, Abreu A, et al Prognostic value of a new cardiopulmonary exercise testing parameter in chronic heart failure: oxygen uptake efficiency at peak exercise - comparison with oxygen uptake efficiency slope. Rev Port Cardiol (Engl Ed) 2011;30:781–7. 10.1016/S0870-2551(11)70026-9 22118129

[22] Guazzi M, Arena R, Halle M, Piepoli MF, Myers J, Lavie CJ. 2016 focused update: clinical recommendations for cardiopulmonary exercise testing data assessment in specific patient populations. Circulation 2016;133:e694–711. 10.1161/CIR.0000000000000406 27143685

[23] Sun XG, Hansen JE, Garatachea N, Storer TW, Wasserman K. Ventilatory efficiency during exercise in healthy subjects. Am J Respir Crit Care Med 2002;166:1443–8. 10.1164/rccm.2202033 12450934

[24] Balmain BN, Tomlinson AR, MacNamara JP, Hynan LS, Wakeham DJ, Levine BD, et al Reducing pulmonary capillary wedge pressure during exercise exacerbates exertional dyspnea in patients with heart failure with preserved ejection fraction. Chest 2023;164:686–99. 10.1016/j.chest.2023.04.003 PMC10548458 37030529

