# Supplementary material for: Left ventricular transmural pressure and ventilatory inefficiency during exercise in advanced heart failure
Source: ESC Heart Fail. 2026 Oct 7;13(5):xvag247. doi: 10.1093/eschf/xvag247 (PMC13644366; doi:10.1093/eschf/xvag247)
Supplement: Supplementary file 1 [file xvag247_supplementary_data.zip › supplementary_figure_legends.docx]

**Supplementary Figure 1. Directed acyclic graph (DAG) illustrating the hypothesized causal structure underlying the association between transmural pressure difference and ventilatory efficiency.**

The exposure (transmural pressure difference, ∆P_TM_) is shown as a solid green node, while green open nodes represent ancestors of the exposure. Pink nodes denote variables that are common causes (ancestors) of both the exposure and the outcome, and the blue node represents the outcome (ventilatory efficiency, V_E_/VCO₂ slope). Arrows indicate assumed causal relationships. DAG-derived confounders included age, sex, body mass index, left ventricular ejection fraction, tricuspid annular plane systolic excursion (TAPSE), glomerular filtration rate, hemoglobin level, chronic obstructive pulmonary disease, atrial fibrillation, pulmonary vascular resistance (PVR) and ischemic etiology, which were selected for multivariable adjustment.

**Supplementary Figure S2. Flow diagram of the study population enrollment.**

Out of 534 patients screened, 112 were excluded based on predefined criteria: 71 patients (63%) with LVEF > 25%, 15 patients (13%) with severe chronic pulmonary disease, and 26 patients (24%) due to incomplete or missing CPET or RHC data. The final study population consisted of 422 patients with advanced systolic heart failure (defined as LVEF ≤ 25%).

**Supplementary Figure S3. Posterior Predictive Check for Model Calibration**

The density distribution plot illustrates the diagnostic validation of the primary Gaussian multivariable Bayesian framework. The thick dark-blue line *(y)* represents the empirically observed kernel density of the baseline V_E_/VCO_2_ slope within the clinical cohort. The cloud of thin light-blue lines (*y_rep_*) represents 100 independent synthetic datasets generated and simulated directly from the posterior predictive distribution. The tight geometric synchronization and overlay between the observed and simulated distributions confirm excellent model calibration, demonstrating that the structural prior configuration successfully captures the empirical variance without systematic bias.
