# Supplementary material for: Left ventricular transmural pressure and ventilatory inefficiency during exercise in advanced heart failure
Source: ESC Heart Fail. 2026 Oct 7;13(5):xvag247. doi: 10.1093/eschf/xvag247 (PMC13644366; doi:10.1093/eschf/xvag247)
Supplement: Supplementary file 1 [file xvag247_supplementary_data.zip › supplementary_material.docx]

**Supplementary Material**

**Model Estimation and Sampling Parameters**

All multivariable regression models in this study were estimated within a generative Bayesian framework using the Stan probabilistic programming language via the brms package (version 2.21.0) in R (version 4.4.0).

To ensure exhaustive and stable exploration of the posterior parameter space, each model was fit using four independent Markov Chain Monte Carlo (MCMC) chains. Each chain was executed for 4,000 iterations, with the first 1,000 draws discarded as a warmup phase to optimize the No-U-Turn Sampler (NUTS) algorithm. This setup yielded a cumulative pool of 12,000 post-warmup draws per model parameter. The target acceptance rate was set to 0.95 to prevent divergent transitions and guarantee numerical stability.

**Prior Distribution Specifications**

Prior distributions were structured to incorporate weak informative regularization, prioritizing a conservative estimation framework that mitigates overfitting and penalizes parameter inflation:

1. **Baseline Clinical Covariates:** For all continuous clinical adjustments (age, BMI, LVEF, TAPSE, eGFR, hemoglobin) and categorical/binary predictors coded as 0–1 (atrial fibrillation, sex, COPD, and ischemic etiology), a symmetrically regularizing prior was applied:

**β_adj_ ~ Normal (0,5)**

This formulation assumes that baseline covariate effects are stable and modest, restricting the model from over-adjusting to sample-specific noise.

2. **Primary Hemodynamic Exposure:** For the primary clinical exposure (ΔP_TM_), an intentionally tighter regularizing constraint was implemented:

**β_hemodynamic_ ~ Normal (0,2)**

3. **Intercept and Residual Error** (**σ):** The regression baseline intercept was specified using a wide, flexible Student-t distribution to accommodate the range of human ventilatory efficiency (VE/VECO_2_ Slope). Residual variance was modeled under a zero-bounded Half-Student-t distribution (σ > 0), allowing the model's error scale to adapt dynamically to the sample variance without arbitrary constraints.

**Convergence and Model Fit Diagnostics**

MCMC chain convergence and mixing were evaluated using peer-reviewed diagnostics. All estimated parameters achieved a potential scale reduction factor (R-hat) of exactly **1.00**, indicating absolute model convergence. The effective sample sizes (**Bulk_ESS** and **Tail_ESS**) routinely exceeded **9,000 independent draws**, confirming that the posterior mass was free from simulation artifacts.

Model calibration was verified via graphical Posterior Predictive Checks, demonstrating that synthetic data replicates generated from the posterior distribution overlay the observed clinical distribution cleanly.

Out-of-sample predictive performance and formal model comparisons were adjudicated using Leave-One-Out Cross-Validation and the Widely Applicable Information Criterion. Epistemic model probabilities were quantified using bridge-sampled Bayes Factors. Finally, to ensure strict framework invariance, all primary Bayesian formulations were replicated and verified against traditional frequentist Ordinary Least Squares regression models using identical covariate adjustment sets.

**Supplementary Table S1. Ordinary Least Square Linear Regression Analysis of VE/VCO₂ Slope**

| **Predictor** | **β (OLS)** | **SE** | **95% CI** | **p value** |
| --- | --- | --- | --- | --- |
| Transmural pressure difference (mmHg) | -0.33 | 0.12 | -0.57 to -0.09 | 0.007 |
| Atrial fibrillation | 2.31 | 1.30 | -0.24 to 4.86 | 0.076 |
| Pulmonary vascular resistance (WU) | -0.18 | 0.39 | -0.95 to 0.59 | 0.65 |
| Female sex | -0.35 | 1.34 | -2.98 to 2.28 | 0.79 |
| Age (years) | 0.06 | 0.10 | -0.13 to 0.25 | 0.53 |
| BMI (kg/m²) | 0.09 | 0.20 | -0.30 to 0.48 | 0.66 |
| LVEF (%) | -0.18 | 0.27 | -0.71 to 0.35 | 0.51 |
| TAPSE (cm) | 0.21 | 1.25 | -2.24 to 2.66 | 0.87 |
| eGFR (mL/min/1.73 m²) | -0.11 | 0.05 | -0.21 to -0.02 | 0.021 |
| Hemoglobin (g/dL) | -2.36 | 0.50 | -3.34 to -1.38 | <0.001 |
| COPD | 0.59 | 1.33 | -2.02 to 3.20 | 0.66 |
| Ischemic etiology | -0.49 | 1.24 | -2.92 to 1.94 | 0.69 |

**Supplementary Table S2. Bayesian multivariable associations between transmural pressure difference and peak VO₂ (mL/kg/min)**

| **Predictor** | **Posterior Mean (β)** | **95% Credible Interval (CrI)** |
| --- | --- | --- |
| **Primary Hemodynamic Exposure** |  |  |
| Transmural Pressure (mmHg) | 0.06 | -0.02 to 0.14 |
| **Adjustment Variables** |  |  |
| Atrial fibrillation | -2.30 | -3.34 to -1.27 |
| PVR (Wood Units) | 0.02 | -0.16 to 0.19 |
| Hemoglobin (g/dL) | 0.73 | 0.51 to 0.94 |
| COPD | -1.48 | -2.66 to -0.29 |
| eGFR (mL/min/1.73m^2^) | 0.03 | 0.01 to 0.05 |
| BMI (kg/m^2^) | -0.08 | -0.17 to -0.00 |
| Ischemic etiology | 0.46 | -0.44 to 1.35 |
| Sex (female) | 0.33 | -0.83 to 1.47 |
| Age (years) | 0.00 | -0.04 to 0.05 |
| LVEF (%) | 0.02 | -0.09 to 0.14 |
| TAPSE (cm) | 0.23 | -0.76 to 1.19 |

**Abbreviations:** BMI: body mass index, COPD: chronic obstructive pulmonary disease, CrI: credible interval, eGFR: estimated glomerular filtration rate, LVEF: left ventricular ejection fraction, TAPSE: tricuspid annular plane systolic excursion, VO_2_: oxygen consumption.

**Supplementary Table S3. Bayesian multivariable associations between conventional filling pressures and peak VO_2_**

| **Model Specification** | **Parameter** | **Posterior Mean (β)** | **95% Credible Interval (CrI)** | **Convergence (R-hat)** | **Bulk_ESS / Tail_ESS** |
| --- | --- | --- | --- | --- | --- |
| **Model A:** *LVEDP Alone* | LVEDP (per mmHg) | 0.00 | -0.07 to +0.07 | 1.00 | 13714 / 8334 |
| **Model B:** *RAP Alone* | RAP (per mmHg) | -0.00 | -0.09 to +0.09 | 1.00 | 13734 / 9022 |
| **Model C:** *Joint Model* | LVEDP (per mmHg)    RAP (per mmHg) | 0.00    -0.00 | -0.09 to +0.10    -0.13 to +0.12 | 1.00    1.00 | 14173 / 9394    13949 / 9005 |

Note: All models are adjusted for AF, PVR, gender, age, BMI, LVEF, TAPSE, eGFR, hemoglobin, COPD, and ischemic etiology.
